# Efficiency of the skeletonized Pendulum K appliance for non-compliance maxillary molar distalization: A clinical pilot study

**DOI:** 10.1007/s00056-021-00280-8

**Published:** 2021-03-02

**Authors:** Gero Stefan Michael Kinzinger, Jan Hourfar, Jörg Alexander Lisson

**Affiliations:** Department of Orthodontics (G56), Saarland University, Kirrberger Straße 100, 66424 Homburg/Saar, Germany

**Keywords:** Anchorage loss, Distal drift, Anchorage, Distal tipping, Rotation, Verankerungsverlust, Distaldrift, Verankerung, Distalkippung, Rotation

## Abstract

**Purpose:**

Conventional anchorage with exclusively intraorally anchored appliances for non-compliance molar distalization combines a palatal acrylic button with periodontal anchorage. This type of anchorage is critically discussed because of the temporary hygienic impairment of the palate and the uncertain anchoring quality of the button. A purely dentally/periodontally anchored Pendulum K appliance was developed, which is exclusively anchored via four occlusal rests. The aims of this pilot study were to examine the suitability of the skeletonized Pendulum K for distalization of maxillary molars, and to investigate the quality of this alternative anchoring modality.

**Patients and methods:**

In all, 10 patients received skeletonized Pendulum K appliances attached to all maxillary premolars for bilateral molar distalization. Supporting anchorage through an acrylic button adjacent to the anterior palate was not used. The pendulum springs were initially activated on both sides with a distalization force of 220 cN each and provided with uprighting and toe-in bends. The specific force/moment system was regularly reactivated intraorally by adjustment of the distal screw.

**Results:**

The study demonstrates the suitability of the skeletonized Pendulum K appliance for the distalization of maxillary molars (3.28 ± 0.73 mm). Side effects on the molars were slight distal tipping (3.50 ± 2.51°/PP, 3.00 ± 1.41°/SN) and mesial inward rotation (average 2.75 ± 7.50° and 4.50 ± 12.77°). Significant anchorage loss occurred in the form of mesialization of the incisors by 1.40 ± 0.82 mm and of the first premolars by 2.28 ± 0.85 mm.

**Conclusion:**

The skeletonized Pendulum K appliance allows compliance-free upper molar distalization. Exclusively dental/periodontal anchorage resulted in a lower percentage of molar distalization compared to a conventional anchoring preparation of the Pendulum K with a palatal acrylic button. Anchorage loss had a comparatively stronger effect on the anchoring premolars but less on the incisors. Typical side effects on the molars such as distal tipping and mesial inward rotation were remarkably low.

## Abstract (de)

**Ziel:**

Konventionelle Verankerungen von ausschließlich intraoral verankerten Apparaturen zur Non-Compliance-Molaren-Distalisierung kombinieren eine palatinale Kunststoffpelotte mit einer parodontalen Verankerung. Diese Art der Verankerung wird wegen der vorübergehenden hygienischen Beeinträchtigung des Gaumens und der unsicheren Verankerungsqualität der Pelotte kritisch diskutiert. Es wurde ein rein dental/parodontal verankertes Pendulum K entwickelt, das ausschließlich über 4 okklusale Auflagen verankert wird. Ziel der vorliegenden Studie war es, die Eignung des skelettierten Pendulum K für die Distalisation von Oberkiefermolaren und die Qualität dieser alternativen Verankerungspräparation zu untersuchen.

**Material und Methoden:**

Bei 10 Patienten wurden zur bilateralen Molarendistalisation skelettierte Pendulum-K-Apparaturen an den ersten und zweiten Prämolaren befestigt. Auf eine unterstützende Verankerung in Form einer dem anterioren Gaumen anliegenden Kunststoffpelotte wurde verzichtet. Die Pendelfedern wurden initial beidseits mit einer Distalisationskraft von jeweils 220 cN aktiviert und mit Aufrichte- und Toe-in-Biegungen versehen. Durch regelmäßiges Verstellen der Distalschraube wurde das spezifische Kräfte‑/Momente-System regelmäßig intraoral reaktiviert.

**Ergebnisse:**

Die Studie belegt die Eignung der skelettierten Pendulum-K-Apparatur zur translatorischen Distalisation der Molaren (3,28 ± 0,73 mm) bei sehr geringen Distalkippungen (3,50 ± 2,51°/PE, 3,00 ± 1,41°/SN) und geringen Mesialeinwärtsrotationen (durchschnittlich 2,75 ± 7,50° bzw. 4,50 ± 12,77°). Die reziprok auf die Verankerungseinheit einwirkenden Kräfte wurden durch die Verankerungseinheit aus 4 Verankerungszähnen abgefangen, wobei signifikante Verankerungsverluste in Form einer Mesialisation der Schneidezähne um 1,40 ± 0,82 mm sowie der ersten Prämolaren um 2,28 ± 0,85 mm feststellbar waren.

**Schlussfolgerungen:**

Die skelettierte Pendulum-K-Apparatur ermöglichte eine kooperationsunabhängige, translatorische Molarendistalisation. Die alleinige Verankerungspräparation aus dem Parodont von 4 Ankerzähnen verursachte einen geringeren prozentualen Anteil der Molarendistalisation an der Gesamtbewegung als ein konventioneller Verankerungsaufbau des Pendulum K mit einer Gaumenpelotte aus Kunststoff. Der reziprok erfolgende Verankerungsverlust wirkte vergleichsweise stärker auf die Prämolaren und somit auf die direkten Ankerzähne, weniger auf die Schneidezähne. Mögliche Nebenwirkungen auf die Molaren wie Distalkippungen und Mesialeinwärtsrotationen fielen jedoch äußerst gering aus.

## Introduction

Upper molar distalization is a possible method for space creation in the dental arch to avoid extraction therapy. Many non-compliance appliances have been described for maxillary molar distalization, including various pendulum appliance types [1–6, 10–12, 14–16, 21–24, 29–31]. The Pendulum K was introduced in 2000 and has proven to be particularly suitable for clinical use [15]. Due to its special biomechanics, this modification of the pendulum appliance enables rapid and friction-free molar distalization, both before and after eruption of the second molars [15, 16, 21–25].

The recommended forces for molar distalization range from 180 to 250 cN per side, depending on the patient’s dentition stage. To avoid undesirable side effects, the reciprocally acting forces and moments must be compensated by an adequate anchoring unit. The conventional anchoring structure of a Pendulum K consists of a combination of dental/periodontal anchoring and an additional intraoral anchoring aid: several maxillary teeth are joined together into an anchoring block through occlusal wire supports together with an acrylic palatal button [15, 16, 21–25].

However, this anchoring modality has its drawbacks: The anchoring effect of palatal buttons made of polymethyl methacrylate is uncertain, and the limited hygienic capability due to the temporary partial covering of the palate is ever present and thus widely discussed [8, 9, 17].

The aim of this pilot study was to test the clinical efficiency of a skeletonized Pendulum K with a solely dental/periodontal anchorage. The extent of anchorage loss in the overall sagittal movement as well as the dental angulation changes and thus quantity and quality of the reduced anchorage preparation in comparison to other, conventionally anchored cooperation-independent pendulum appliances will be discussed regarding the literature. In addition, a comparison to other, conventional intraorally anchored non-compliance pendulum appliances will be made.

## Materials and methods

### Patients

All patients received treatment in an orthodontic specialist practice by exclusively one orthodontist (G.K.) over a period of 12 months. The patients required bilateral maxillary molar distalization due to a dentoalveolar class II occlusion including an arch length discrepancy with substantial loss of E‑space in the maxillary dentition.

The patients could choose between three different anchorage options for a Pendulum K appliance after being shown pictures of each: conventional with Nance pad, purely skeletal with mini screws or purely dental/periodontal. In all, 10 patients with a mean age of 13 years and 4 months (7 girls, 3 boys) opted for treatment with a skeletonized Pendulum K appliance.

The average treatment duration was 17.2 weeks. Out of 20 second molars, 10 had already reached the occlusal plane, 4 were erupting and 6 were still impacted.

### Skeletonized Pendulum K

The skeletonized Pendulum K used in this pilot study has no palatal acrylic button as an anchoring element. A relatively new, dedicated distalization screw (order # A167D1639, Forestadent, Pforzheim, Germany) is the basis for the appliance. It consists of a lasered composite of two 1.5 mm-thick wires and a distal screw. There is a holding device attached to the body of the distal screw for accepting the pendulum springs, which are individually made of beta titanium wire. The two laser-cut round metal rods are adapted three-dimensionally “butterfly-winged” to the specific palatal arch of the patient on the working model and connected to the occlusal rests, which are also individually manufactured from 1 mm spring-hard wire, to form a purely dental/periodontal support. The pendulum springs are manufactured similarly as in the conventional Pendulum K, but for both sides the pendulum springs are manufactured from one piece of TMA (titanium molybdenum alloy) wire. The connecting part is designed as a double retention part so that it can be inserted into the small lock attached to the screw.

**Fig. 1:**
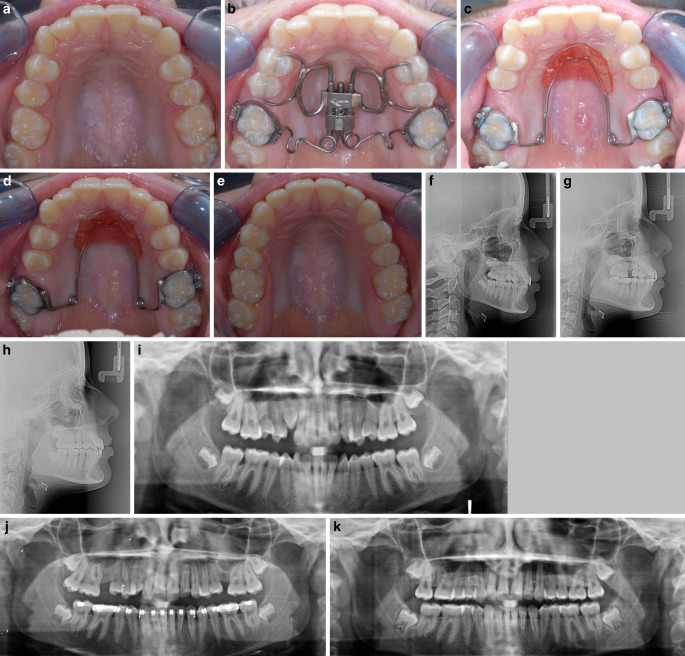
Skeletonized Pendulum K appliance, dental anchorage only. Patient example: female patient 14 years, 9 months of age, duration of molar distalization treatment 24 weeks. **a** Pretreatment: bilateral mesial migration of cuspids, premolars and molars. **b** Occlusal view immediately after skeletonized Pendulum K placement. **c** Occlusal view after completion of molar distalization: clinical assessment reveals bodily molar distalization. Retention using a combination of reduced Nance button and bi-helix. **d** Distal drift of cuspids and premolars after molar distalization und tendency of “self-alignment” of the dental arch. **e** Posttreatment: well-aligned dental arch. **f–h** Lateral cephalograms and **i–k** orthopantomograms at pretreatment, during treatment and posttreatment demonstrate bodily molar distalization Skelettierte Pendulum-K-Apparatur, nur dentale Verankerung. Patientenbeispiel: Patientin, 14/9 Jahre alt, Dauer der Molarendistalisationsbehandlung 24 Wochen. **a **Vor der Behandlung: bilaterale Mesialwanderung der Eckzähne, Prämolaren und Molaren. **b **Okklusalansicht unmittelbar nach Einsetzen der skelettierten Pendulum-K-Apparatur. **c **Okklusalansicht nach Abschluss der Molarendistalisation: Bei der klinischen Beurteilung zeigt sich eine körperliche Molarendistalisation. Retention mit einer Kombination aus reduziertem Nance-Pelotte und Bi-Helix. **d **Distaldrift der Eckzähne und Prämolaren nach Molarendistalisation und Tendenz zur „ Selbstausrichtung“ des Zahnbogens. **e** Nach der Behandlung: gut ausgeformter Zahnbogen. **f–h **Fernröntgenseitenbilder und **i–k **Orthopantomogramme vor der Behandlung, während und nach der Behandlung zeigen eine körperliche Molarendistalisation

All components of the skeletonized Pendulum K are made of metal. The appliance has no direct contact with the mucosa. This enables the patient to clean and rinse both the appliance and the palatal mucosa properly (Fig. 1a–k).

The preactivation of the two pendulum springs with distalization force, uprighting activation and toe-in bending is performed on the working model as in the classic version [18, 23, 26]. The reactivation of the specific force/torque system is achieved through adjusting the incorporated distal screw at 4‑week intervals.

Both cast and cephalometric analyses have been described and used by Kinzinger et al. [27] in a previous study of a different, but related appliance. We have used their methodology to allow comparability of the results.

### Cast analysis

**Fig. 2:**
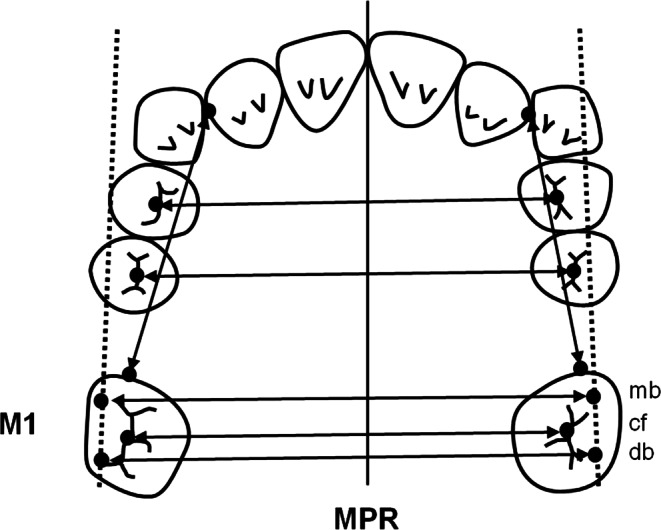
Cast analysis (changes in the horizontal plane): angular and linear measurements conducted to determine changes in the transverse width of the dental arch and rotation at the first molars Modellanalyse (Veränderungen in der horizontalen Ebene): Durchführung von Winkel- und Streckenmessungen zur Bestimmung von Veränderungen in der transversalen Breite des Zahnbogens und der Rotation an den ersten Molaren

Dental plaster casts taken at the start of treatment (T1) and after Pendulum K appliance removal (T2) were analyzed to investigate molar movement in the horizontal plane. Analysis objectives were changes in length of the supporting zone, potential increase or decrease of arch width at premolars and first molars, and the extent and kind of first molar rotation. The distance between the distal point of contact of the lateral incisor and the mesial point of contact of the first molar, bilaterally, the distance between the lowest point of the central fossa (cF), the mesiobuccal (mb) and the distobuccal (db) cusps of the first molar were registered for every cast. In addition, the angles between a line running through the mesiobuccal (mb) and distobuccal (db) cusps and the midpalatal raphe (MPR) were measured (Fig. 2).

### Cephalometric analysis

**Fig. 3:**
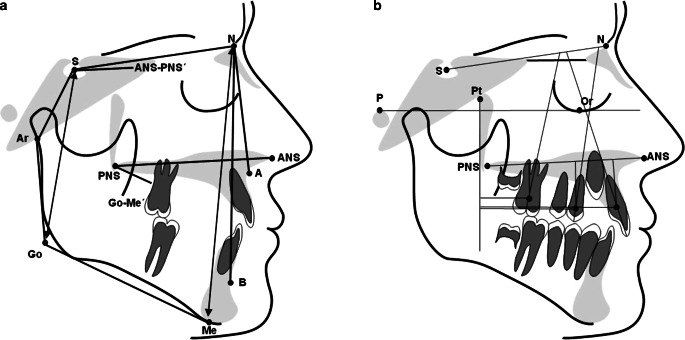
Cephalometric analysis (changes in the sagittal plane): angles and distances registered on the lateral cephalogram before and after molar distalization. **a** Skeletal angular and linear measurements. **b** Dental angular and linear measurements Kephalometrische Analyse (Veränderungen in der Sagittalebene): auf dem Fernröntgenseitenbild gemessene Winkel und Längen vor und nach der Molarendistalisation. **a** Skelettale Winkel- und Streckenmessungen. **b** Dentale Winkel- und Streckenmessungen

Lateral cephalograms recorded at the start of treatment (T1) and on completion of distalization (T2) were traced to determine changes of the following parameters (Fig. 3).

- SNA = angle between the anterior cranial base and the deepest point of the ventral concavity of the maxilla
- SNB = angle between the anterior cranial base and the deepest point of the ventral concavity of the mandible
- ANB = angle between the deepest point of the ventral concavity of the maxilla and the deepest point of the ventral concavity of the mandible
- S‑N/Go-Me = angle between the anterior cranial base and the mandibular plane
- S‑N/ANS-PNS = angle between the anterior cranial base and the palatal plane
- ANS-PNS/Go-Me = angle between the palatal plane and the mandibular plane
- Björk’s summation angle = sum of saddle angle (NSAr), articular angle (SArGo), and gonion angle (ArGoMe)
- S‑Go:N-Me = facial height ratio: posterior face height to anterior face height
- U1-CEJ/PTV = distance from maxillary central incisor to pterygoid vertical
- U4-CEJ/PTV = distance from maxillary first premolar to pterygoid vertical
- U6-CEJ/PTV = distance from maxillary first molar to pterygoid vertical
- U1/ANS-PNS = angle between maxillary central incisor and palatal plane
- U1/SN = angle between maxillary central incisor and anterior cranial base
- U4/ANS-PNS = angle between maxillary first premolar and palatal plane
- U4/SN = angle between maxillary first premolar and anterior cranial base
- U6/ANS-PNS = angle between maxillary first molar and palatal plane
- U6/SN = angle between maxillary first molar and anterior cranial base
- U1-CEJ/ANS-PNS = distance from maxillary central incisor to palatal plane
- U4-CEJ/ANS-PNS = distance from maxillary first premolar to palatal plane
- U6-CEJ/ANS-PNS = distance from maxillary first molar to palatal plane

The angles between anterior cranial base and A landmark (SNA), anterior cranial base and B landmark (SNB), and A landmark and B landmark (ANB), the angle between the anterior cranial base and the mandibular plane, the angle between the anterior cranial base and the palatal plane, the angle between the palatal plane and the mandibular plane, Björk’s summation angle, and the facial height ratio were measured or computed to verify any skeletal changes.

In the sagittal plane, the relative incisor and first premolar mesial movement, hence the anchorage loss, as well as the relative first molar distal movement in relation to the pterygoid vertical (U1-CEJ/PTV; U4-CEJ/PTV; U6-CEJ/PTV) were determined. The respective points of reference for the measurements were the cementoenamel junction (CEJ) found on the longitudinal axis of the teeth. Growth-induced changes (increase by 1 mm per year) were taken into account in analogy to the Ricketts’ analysis.

The extent of mesial tipping of the incisors and first premolars and of distal tipping of the first molars was determined based on the angles between the longitudinal tooth axis and, respectively, the palatal plane or the anterior cranial base (U1/ANS-PNS, U1/SN; U4/ANS-PNS, U4/SN; U6/ANS-PNS, U6/SN).

Potential tooth intrusions and extrusions were verified in relation to the palatal plane (U1-CEJ/ANS-PNS, U4-CEJ/ANS-PNS, U6-CEJ/ANS-PNS).

All linear measurements were carried out with a digital caliper gauge (Burg-Wächter PRECISE PS 7215, measuring accuracy 0.01 mm, Burg-Wächter, Wetter, Germany). The angular measurements were performed using a dedicated tracing software (fr-win®, Computer konkret AG, Falkenstein, Germany) (measuring accuracy 0.1°).

### Statistical analysis

Statistical computations were performed using SPSS® 14 (IBM, Armonk, NY, USA). Casts and lateral cephalograms were traced twice with a 4-week interval in each case. If values were found to deviate, the mean of both measurements was fed into the statistical analysis. Then, the arithmetic mean and the standard deviation were computed for every variable used in the in vivo measurements and statistical analysis of the changes of individual variables from start of treatment (T1) to Pendulum K appliance removal (T2) was performed by a one-sample t‑test. It was checked thereby which effective changes therapeutically induced by the treatment were evident against the null hypothesis. Differences for which the probability of error was less than 5% (*p* < 0.05) were considered statistically significant.

Study casts and lateral cephalograms taken before and after the pendulum appliance therapy were measured or traced and evaluated twice at an interval of 3 months. The method error (ME) was then calculated using the Dahlberg formula (ME = √(Σd^2^/2n)) [7]. The overall ME of the various measurements used in this study was no greater than 0.72 mm for linear and 0.68° for angular measurements. The ME was < 1 for all measurements.

Due to the limited number of patients of this pilot study, *P*-values should be interpreted rather descriptive than confirmatory.

## Results

### Dental cast analysis

Maxillary cast measurements before and after molar distalization with a skeletonized Pendulum K appliance revealed the first molar position changes (Table 1).

**Table 1:** Changes in first upper molar position induced by skeletonized Pendulum K therapy in the horizontal plane (cast analysis) Veränderungen der Position des ersten oberen Molaren durch die Behandlung mit dem skelettierten Pendulum K in der horizontalen Ebene (Modellanalyse)

Cast analysis	*N*	T1 M T1 SD		T2 M T2 SD		∆T1-T2 M ∆T1-T2 SD		Significance	*p*-value
UR2 distal − UR6 mesial (mm)	10	21.55	1.47	26.10	0.91	−4.55	0.79	*	0.001
UL2 distal − UL6 mesial (mm)	10	21.20	2.76	25.85	1.73	−4.65	1.74	*	0.013
Central fossa (cF) UR4 − UL4 (mm)	10	34.00	2.25	34.08	2.30	−0.10	0.14	ns	0.391
Central fossa (cF) UR5 − UL5 (mm)	10	39.43	2.83	39.45	2.85	−0.03	0.05	ns	0.391
Mesiobuccal cusp tips (mb) UR6 − UL6 (mm)	10	51.40	3.01	51.90	3.48	−0.50	2.50	ns	0.716
Central fossa (cF) UR6 − UL6 (mm)	10	46.48	2.36	47.25	2.16	−0.78	0.99	ns	0.216
Distobuccal cusp tips (db) UR6 − UL6 (mm)	10	54.33	2.79	55.55	2.55	−1.23	0.55	*	0.016
UR6 rotation (°)	10	18.88	9.44	21.63	14.78	−2.75	7.50	ns	0.516
UL6 rotation (°)	10	10.75	4.19	15.25	11.84	−4.50	12.77	ns	0.523

Determination of type of molar rotation: angle between midpalatal raphe (*MPR*) and a line running through the mesiobuccal and distobuccal cusps of the molars; for ∆T1–T2 (value before distalization) − (value after distalization): positive value = mesiobuccal and distopalatal rotation, negative value = mesiopalatal or distobuccal rotation

*N* number of measurements, *M* mean, *SD* standard deviation, *ns* not significant

*P < 0.05; **P < 0.01; ***P < 0.001

The supporting zones increased by 4.55 ± 0.79 mm in the first quadrant and by 4.65 ± 1.74 mm in the second quadrant. The transverse width of the dental arch decreased by 0.50 ± 2.50 mm between the mesiobuccal cusps, by 0.78 ± 0.99 mm between the central fossae, and by 1.23 ± 0.55 mm between the distobuccal cusps. In addition, the first molars have rotated mesiopalatally and distobuccally in the first quadrant by 2.75 ± 7.50°, and in the second quadrant by 4.50 ± 12.77°. Thus, the increase of the supporting zones was significant.

### Cephalometric analysis

Cephalometrics showed that the cranial base remained constant, with a change of the SNA angle of only 0.65 ± 1.51° and the SNB angle of only 0.55 ± 1.71°. The positional relationship of the palatal plane to the anterior cranial base and to the mandibular plane was virtually unchanged. Björk’s summation angle changed by only 0.80 ± 1.75° in the course of molar distalization. All changes of skeletal parameters occurring between T1 and T2 were not significant (Table 2).

**Table 2:** Skeletal angular and linear measurements (cephalometric analysis) Skelettale Winkel- und Streckenmessungen (kephalometrische Analyse)

Cephalometric analysis	*N*	T1 M	T1 SD	T2 M	T2 SD	∆T1–T2 M	∆T1–T2 SD	Significance	*p*-value
*Skeletal—angular*									
SNA (°)	10	81.75	4.07	81.10	3.51	0.65	1.51	ns	0.452
SNB (°)	10	78.20	3.81	77.65	2.75	0.55	1.71	ns	0.565
ANB (°)	10	3.55	0.97	3.43	0.92	0.13	0.26	ns	0.412
S‑N/Go-Me (°)	10	28.30	4.35	29.10	3.15	−0.80	1.75	ns	0.428
S‑N/ANS-PNS (°)	10	5.15	2.84	6.88	5.04	−1.73	2.36	ns	0.240
ANS-PNS/Go-Me (°)	10	23.18	4.94	22.18	5.79	1.00	2.26	ns	0.441
Björk’s summation angle (°)	10	388.30	4.35	389.10	3.15	−0.80	1.75	ns	0.428
*Skeletal—linear*									
S‑Go:N-Me (%)	10	68.93	3.63	68.93	2.62	0.00	1.54	ns	1.000

*N* number of measurements, *M* mean, *SD* standard deviation, *ns* not significant

*P < 0.05; **P < 0.01; ***P < 0.001

In the area of the cementoenamel junction, the first molars were distalized by 3.28 ± 0.73 mm and intruded by 0.62 ± 1.79 mm. Furthermore, distal tipping by 3.50 ± 2.51° in relation to the palatal plane and by 3.00 ± 1.41° in relation to the anterior cranial base was observed.

The first premolars, which were included in the anchorage setup, mesialized by 2.28 ± 0.85 mm, extruded by 0.45 ± 0.37 mm, and tipped by 1.25 ± 3.69° in relation to the palatal plane, and by 1.00 ± 2.16° in relation to the anterior cranial base. The central incisors were protruded by 1.40 ± 0.82 mm and were extruded by 0.10 ± 0.08 mm while they showed labial tipping of 2.75 ± 1.89° in relation to the palatal plane and 3.25 ± 2.75° to the anterior cranial base.

The extent of all linear dental movements in relation to the pterygoid vertical was significant (Table 3).

**Table 3:** Dental angular and linear measurements (cephalometric analysis) Dentale Winkel- und Streckenmessungen (kephalometrische Analyse)

Cephalometric analysis	*N*	T1 M	T1 SD	T2 M	T2 SD	∆T1–T2 M	∆T1–T2 SD	Significance	*p*-value
*Dental—angular*									
U1/ANS-PNS (°)	10	106.25	5.32	109.00	6.68	−2.75	1.89	ns	0.062
U1/SN (°)	10	99.50	3.11	102.75	5.44	−3.25	2.75	ns	0.099
U4/ANS-PNS (°)	10	91.50	4.80	92.75	3.30	−1.25	3.69	ns	0.546
U4/SN (°)	10	85.00	1.41	86.00	2.16	−1.00	2.16	ns	0.423
U6/ANS-PNS (°)	10	80.00	7.62	76.50	6.56	3.50	2.51	ns	0.069
U6/SN (°)	10	72.75	6.60	69.75	6.02	3.00	1.41	ns	0.066
*Dental—linear*									
U1-CEJ/PTV (mm)	10	54.08	3.73	55.48	4.22	−1.40	0.82	*	0.043
U4-CEJ/PTV (mm)	10	40.30	4.61	42.58	5.22	−2.28	0.85	*	0.013
U6-CEJ/PTV (mm)	10	23.08	3.04	19.80	3.36	3.28	0.73	**	0.003
U1-CEJ/ANS-PNS (mm)	10	18.43	0.21	18.53	0.25	−0.10	0.08	ns	0.092
U4-CEJ/ANS-PNS (mm)	10	17.38	0.90	17.83	0.56	−0.45	0.37	ns	0.093
U6-CEJ/ANS-PNS (mm)	10	14.83	1.44	14.20	1.79	0.62	1.79	ns	0.087

*N* number of measurements, *M* mean, *SD* standard deviation, *ns* not significant

*P < 0.05; **P < 0.01; ***P < 0.001

The total movements in the sagittal plane amounted to 4.68 ± 0.99 mm for molar distalization and central incisor protrusion together or 5.56 ± 1.21 mm for molar distalization and first premolar mesialization together. Based on a distalization of first molars of 3.28 ± 0.73 mm, molar distalization accounted for 70.78 ± 13.85% and 59.45 ± 9.59%, respectively, of the total movement (Table 4).

**Table 4:** Share of maxillary molar distalization in total sagittal movement (cephalometric analysis) Anteil der Distalisation der Oberkiefermolaren an der Bewegung in der Sagittalebene (kephalometrische Analyse)

Cephalometric analysis	*N*	∆ T1–T2 M	∆ T1–T2 SD
*Dental-linear (mm)*			
U1-CEJ/PTV (mm)	10	−1.40	0.82
U4-CEJ/PTV (mm)	10	−2.28	0.85
U6-CEJ/PTV (mm)	10	3.28	0.73
Total sagittal movement 1–6^a^	10	4.68	0.99
Total sagittal movement 4–6^b^	10	5.56	1.21
*Calculation of ratio (%)*			
Share of molar distalization in total sagittal movement 1–6^c^	10	70.78	13.85
Share of molar distalization in total sagittal movement 4–6^d^	10	59.45	9.59

*N* number of measurements, *M* mean, *SD* standard deviation

^a^Total movement in the sagittal plane 1–6 = [U1 − CEJ/PTV] + [U6 − CEJ/PTV]

^b^Total movement in the sagittal plane 4–6 = [U4 − CEJ/PTV] + [U6 − CEJ/PTV]

^c^Calculation: share of molar distalization in total sagittal movement 1–6 = 100 × (U6 − CEJ/PTV)/ ([U1 − CEJ/PTV] + [U6 − CEJ/PTV])

^d^Calculation: share of molar distalization in total sagittal movement 4–6 = 100 × (U6 − CEJ/PTV)/ ([U4 − CEJ/PTV] + [U6 − CEJ/PTV])

## Discussion

The results show the clinical efficiency of the skeletonized Pendulum K. Both casts and corresponding lateral cephalograms of all patients were measured. The cast findings reflect the clinical findings: The increase of the E‑space occurred as a summation effect of molar distalization and anchorage loss. Only the cephalometric analysis determines both the extent of molar distalization and the anchorage loss as a net effect of the overall sagittal movement.

The measurement of casts showed that, in addition to a support zone extension, a therapeutically desired transverse arch expansion was only achieved with slight mesial inward or distal outward rotation of the molars. A toe-in bend to compensate for the force application palatal from the center of resistance of the molar was placed before the appliance was inserted, and appeared to be as efficient as that described for the classic Pendulum K variant. The anchorage of the pendulum spring retentions in the acrylic button is more rigid than the plugged attachment of the pendulum spring retention in the lock on the screw body in the skeletal appliance, but with the appropriate clamping adhesion, this seems to be sufficient to absorb the reciprocal moments.

The evaluation of the lateral cephalograms showed that the first molars experienced minimal distal tipping. In comparison with other studies regarding pendulum appliances ([1–6, 10, 11, 14–16, 21, 22, 24, 29–31]; Table 5), it becomes obvious that the biomechanics of the Pendulum K, regardless of the form of anchorage, provides the best results in terms of molar distalization: the extent of distal tipping is lowest due to the uprighting activation, which is periodically reactivated by adjusting the distal screw. In the current study, however, the causal factor for bodily molar distalization may also be that in most patients the second molars had largely erupted. Kinzinger et al. [21] have shown in a clinical study with pendulum appliances that the distal tipping of the first molars was comparatively lower when the second molars are already fully erupted. This phenomenon can be explained: Second molars in the germinal stage act like a hypomochlion for the six-year molar to be distalized; thus, the first molar experiences a tipping via the germ of the second molar during its distalization. With increasing root development and eruption of the second molar into the dental cavity, the contact point between the molars shifts continuously to coronal and the tendency to crown tipping of the first molar is reduced.

**Table 5:** Studies using different conventionally intraorally anchored pendulum appliances for maxillary molar distalization: distal tipping of molars (°), molar distalization (%) and anchorage loss in total movement (%) Studien mit verschiedenen konventionell intraoral verankerten Pendelapparaturen zur Oberkiefermolarendistalisation: distale Kippung der Molaren (°), Molarendistalisation (%) und Verankerungsverlust in der Gesamtbewegung (%)

Type of pendulum appliance/author(s) and reference	Treatment cases	Dental anchorage of the pendulum appliance used	Soft tissue support	Distal tipping of molars (°)	Share of molar distalization in total movement (%)	Share of anchorage loss in total movement (%)
*Hilgers pendulum*						
Gosh and Nanda [11]	41	Hilgers pendulum 4 OW	NP	8.36 ± 8.37 SN	56.9 PM1	43.1 PM1
Byloff and Darendeliler [3]	13	Hilgers pendulum 4 OW	NP	14.50 ± 8.33 PP	70.9 PM1	29.1 PM1
Joseph and Butchart [14]	7	Hilgers pendulum 4 OW	NP	15.7 PP	57.9 I	42.1 I
Bussick and McNamara Jr [2]	101	Hilgers pendulum 4 OW	NP	10.60 ± 5.60 FH	76.0 PM1	24.0 PM1
Toroglu et al. [31]	14	Hilgers pendulum 4 OW	NP	14.9 ± 5.3 FH	55.1 PM2 73.7 I	44.9 PM2 26.3 I
Toroglu et al. [31]	16	Hilgers pendulum 4 OW	NP	13.4 ± 4.6 FH	38.3 PM2 50.0 I	61.7 PM2 50.0 I
Chaques-Asensi and Kalra [5]	26	Hilgers pendulum 2 B PM1	NP	13.06 ± 7.52 SN	70.6 PM1 71.8 I	29.4 PM1 28.2 I
Chiu et al. [6]	32	Hilgers pendulum 4 OW	NP	10.7 ± 5.5 FH	81.0 PM	19.0 PM
Fuziy et al. [10]	31	Hilgers pendulum 2 B PM1 2 OW PM2	NP	18.57 ± 3.0 FH	63.5 PM1	36.5 PM1
Öncag et al. [29]	15	Hilgers pendulum 2 B PM1 2 OW PM2	NP	6.01 ± 2.84 SN	57.1 PM1 71.1 I	42.9 PM1 28.9 I
Patel et al. [30]	20	Hilgers pendulum 2 B PM1 2 OW PM2	NP	10.00 ± 4.04 SN	61.1 PM2 73.0 I	38.9 PM2 27.0 I
*Hilgers pendulum with uprighting activation*						
Byloff et al. [4]	20	Hilgers pendulum with uprighting activation 4 OW	NP	6.07 ± 5.15 PP	64.2 PM1	35.8 PM1
Angelieri et al. [1]	22	Hilgers pendulum with uprighting activation 2 B PM1 2 OW PM2	NP	9.4 PP	35.7 PM1 45.4 I	64.3 PM1 54.6 I
*Pendulum K*						
Kinzinger et al. [15]	50	Pendulum K 4 OW	NP	3.24 ± 4.28 SN 3.14 ± 3.99 PP	72.5 I	27.5 I
Kinzinger et al. [16]	20	Pendulum K 4 OW	NP	5.18 ± 3.15 SN	70.3 I	29.7 I
Kinzinger et al. [21]	36	Pendulum K 4 OW	NP	3.07 ± 4.02 SN 3.29 ± 4.31 PP	70.2 I	29.8 I
Kinzinger et al. [22]	30	Pendulum K 4 OW	NP	4.65 ± 3.45 SN 4.18 ± 3.36 PP	76.3 PM1 74.2 I	23.7 PM1 25.8 I
Kinzinger et al. [24]	66	Pendulum K 4 OW	NP	4.24 ± 4.67 SN 4.75 ± 4.50 PP	73.5 I	26.5 I
Kinzinger et al., this study	10	Skeletonized Pendulum K 4 OW	–	3.00 ± 1.41 SN 3.50 ± 2.52 PP	59.4 PM1 70.8 I	40.6 PM1 29.2 I

*SN* anterior cranial base, *FH* Frankfort horizontal, *PP* palatal plane, *I* central incisor, *PM1* first premolar, *PM2* second premolar, *NP* Nance button, *B* premolar bands anchored to the Nance pad using connecting wires, *OW* occlusal wire rests anchored to the Nance button

The conventional anchoring structure of intraorally anchored appliances for cooperation-independent molar distalization in the upper jaw is combined in the form of an acrylic button adjacent to the palatal mucosa and the periodontium of anchor teeth. Disadvantages of this anchorage preparation are particularly limited hygiene [17], and there are contraindications based on certain dentition stages and local findings [24]. In addition, it must be accepted that the anchoring effect of the anterior palatal plate according to Nance on the resilient palatal mucosa is possibly based only on hydrodynamic interactions and is by no means a stationary anchorage. Thus, its anchoring value should not be overestimated [8, 9]. Nevertheless, the results of the present study indicated that purely dental/periodontal anchoring has a reduced anchoring quality: The percentage of molar distalization in the overall sagittal movement, measured in relation to the first premolars integrated in the anchoring preparation, was 59.4% and thus lower than with the conventional Pendulum K appliance variant ([15, 16, 21, 22, 24]; Table 5). In the incisor area, however, anchorage loss was not increased.

Comparison with the literature showed that only half of the studies investigating the Hilgers pendulum including a Nance button showed less anchorage loss in the premolar region than the skeletonized Pendulum K without Nance button in the current study. The other half showed even more anchorage loss (Table 5). In the incisor region, the anchorage loss was comparatively unremarkable. Thus, even with the skeletonized Pendulum K with a purely dental/periodontal anchoring structure, sufficient molar distalization could be achieved.

It should be noted that anchorage loss is not always disadvantageous: especially in Class-III-patients, it can be therapeutically beneficial through providing positive effects for camouflage treatment [19]. However, if an anchorage loss must be avoided during therapy, for example due to specific local contraindications [24], a further option is to use a skeletally anchored version of the Pendulum K [13, 17, 28].

After distalization, the molars must be retained in the therapeutically achieved position. Even if, as in the patients of the present study, an acrylic button was absent during the distalization phase, it should be used as part of a Nance holding arch in the subsequent stabilization phase for a defined period.

Furthermore, it is important to manage the space gained mesial of the distalized molars. In another clinical pilot study, Kinzinger et al. [20] observed and described the distal drift of premolars. The premolars, which were previously reciprocally mesialized as anchor teeth, migrated spontaneously and without force application distally due to tension of the transseptal fibers. No active distal movement of premolars using molar anchorage should be initiated, since the molars are still unstable in their new position. This would inevitably lead to a reactive forward movement of the molars. It is preferable to await a distal drift of the premolars and partially also of the canines prior to treating the entire dental arch with a multibracket appliance (Fig. 1d). The basic prerequisite for sufficient distal drifting is an almost translational, bodily molar distalization. Distally directed premolar movement may only be expected if the space gained by distalization in the apical region is similarly large as in the coronal region [20].

The results of this pilot study show interesting tendencies despite a small number of patients: The purely dental/periodontal anchoring variant has basically proven itself in clinical application and, due to its special biomechanics, created bodily molar distalization.

Further comparative studies with a sufficient number of patients and involving different dentition stages will have to show to what extent anchorage loss differs between the skeletonized appliance and the Nance pad variant.

## Conclusions

The skeletonized Pendulum K appliance without Nance button has proven clinically effective. It allowed compliance-free bodily molar distalization. The amount of distalization appears to be lower when compared with a conventional anchoring abutment including a Nance button. The anchorage loss had a stronger effect on the premolars and thus on the anchor teeth, but less so on the incisors. Typical side effects on molars such as distal tipping and mesial inward rotation were remarkably low.
